# Non-biochemical Risk Factors for Cardiovascular Disease in General Clinic-based Rural Population of Bangladesh

**DOI:** 10.2188/jea.14.63

**Published:** 2005-03-18

**Authors:** M. Mostafa Zaman, Sohel Reza Choudhury, Jasimuddin Ahmed, Sharker Md. Numan, Md. Sadequl Islam, Nobuo Yoshiike

**Affiliations:** 1 National Center for Control of Rheumatic Fever and Heart Diseases, Dhaka, Bangladesh.; 2 Department of Community Medicine, School of Medical Sciences, Universiti Sains Malaysia.; 3 School of Science and Technology, Bangladesh Open University.; 4 Al Raji Hospital, Dhaka, Bangladesh.; 5 National Institute of Health and Nutrition.

**Keywords:** Bangladesh, clinic-based surveys, cardiovascular diseases, risk factors

## Abstract

BACKGROUND: Strategies for preventing premature cardiovascular disease include measures to control its risk factors. To plan such activities, prevalence of these factors must be known. Data regarding risk factor prevalence is limited in Bangladesh and measurement of biochemical factors is no always feasible. The aim of our study is to describe the non-biochemical risk factors in a clinic-based rural population of Bangladesh that would reflect at least a part of the problem in the rural area.

METHODS: A cross sectional study was done in a clinic based patient population aged 20 years and older (471 males and 800 females) in a rural community of Bangladesh. A questionnaire on lifestyle including dietary and smoking habit was administered and physical examinations including height, weight, waist circumference, and blood pressure were measured in standardized way.

RESULTS: Mean body mass index was 18.5 kg/m^2^ (standard deviation [SD]: 2.9 kg/m^2^) in males and 18.7 kg/m^2^ (SD: 3.3 kg/m^2^) in females. Mean systolic blood pressure was 120.0 mmHg (SD: 18.5 mmHg) and mean diastolic blood pressure 77.2 mmHg (SD: 9.9 mmHg) in all subjects. The prevalence of hypertension (140+/90+ mmHg and/or on treatment) was 17.8%. Prevalence of tobacco consumption (smoking and chewing) was 43.8% in males and 27.1 in females. Prevalence of abdominal obesity (waist circumference >94 cm in males, >80 cm in females) was 1.6 % and 11.4 % for males and females respectively. Proportion of overweight (BMI 25.0+) was 3.6%.

CONCLUSION: Prevention programs and measures should be emphasized for the control of tobacco and hypertension in general, and central obesity in females, as far as rural population of Bangladesh is concerned.

Cardiovascular disease (CVD) is rapidly emerging as an important cause of mortality and morbidity in developing countries.^1^ Epidemiologic studies done in various populations have identified major risk factors for CVD, which include both biochemical and non-biochemical factors such as inappropriate diet and physical activity leading to high body mass index (BMI), raised blood pressure, tobacco use, unfavorable blood lipid, and impaired glucose tolerance. One report suggested that more than 75 % of CVD in population could be explained by the prevalence of these established risk factors.^2^ Strategies for preventing premature CVD include measures to control its major risk factors. Measurement of biochemical factors is not always feasible in developing countries due to budget constrains and lack of CVD epidemiologic research. However, measurement of established non-biochemical risk factors such as blood pressure, BMI, and smoking can be done in a standardized but less expensive way. Surveillance of these non- biochemical risk factors is important as these risk factors may explain a large part of CVD in developing countries^3^ in which mean energy intake and fat intake is still at lower spectrum. Prevention activities can be initiated on the basis of non-biochemical risk factors, if their prevalences are known. Data on these factors are still inadequate in Bangladesh. A few studies have reported prevalence of individual risk factors such as hypertension, smoking and dietary habit, from urban and rural population located close to Dhaka city.^4^^,^^5^^,^^6^ It needs further corroborations to get a better idea of the situation in rural population.

The aim of our study is to describe the non-biochemical risk factors in a clinic-based rural population of Bangladesh that could be made easily available and would reflect at least a part of the problem in the rural area.

## METHODS

This study was conducted between September 1999 and August 2001 in Ekhlaspur Center of Health situated in Ekhlaspur, Matlab *thana* (sub-district) of Chandpur district, about 60 km southeast of Dhaka city. The population of Ekhlaspur aged 20 years and older is 2,730 (males:1,460, females:1,270). It is an agricultural area and people endure hard physical work mostly as farmer and eat rice and vegetables almost three times a day. Almost all are Muslim by religion and alcohol drinking is unacceptable and remains a taboo to be interviewed. Females usually do not smoke but chew tobacco leaf with betel quid (*pan*).

Subjects are the patients from Ekhlaspur and neighboring villages who had attended the outpatient clinic of the Ekhlaspur Center of Health. Records were kept for all patients who visited the clinic but this analysis was done for those aged 20 years and older (total: 1,271, males: 471, and females: 800) after excluding pregnant females. Only two males and one female were taking medication for hypertension.

Assessment of risk factors by a trained health assistant comprised of administration of a questionnaire on age, sex, education, meat and fish consumption, smoking including chewing tobacco, current drug treatment for chronic diseases, and measurements of weight and height (without heavy clothing and shoes) and waist circumference. For smoking status, patients were asked whether he or she is a current smoker. In case of a smoker, the number of cigarettes smoked per day was recorded. Patients were also asked about the current use of any type of chewing tobacco. All the patients had their blood pressure measured by physicians using mercury sphygmomanometers in sitting position after a 2-minutes rest. Korotkoff phase V was taken as diastolic blood pressure (DBP). Five physicians who had proper training for blood pressure measurement in epidemiologic studies measured blood pressure during their respective consultation days.

Data was analyzed using SAS^®^ (version 6.11 Cary NC, USA). Median and inter-quartile range (IQ) are calculated for variables perceived not to distribute normally. Mean and standard deviation (SD) are given for the normally distributed variables. Percentage and standard error (SE) were calculated for frequency variables. Hypertension was defined as systolic blood pressure (SBP) 140 mmHg or DBP 90 mmHg or higher or on anti-hypertensive drug. Body mass index (BMI) was calculated as weight in kilograms (kg) divided by the square of the height in meters (m^2^).

## RESULTS

Among the total number of patients aged 20 years and older visiting the center between September 1999 and August 2001 who had been diagnosed with a specific disease, the highest number had peptic ulcer diseases (total: 14.3%, males: 12.3 %, and females: 15.5% [Table 1]). Of the total patients, 5.1% had diagnosis of hypertension while 1.2 % had diagnosis of ischemic heart disease. However, only two patients with primary diagnosis of stroke were seen in the clinic during the study period, which means that mainly ambulatory patients received treatment from our center.

**Table 1.** Primary* diagnoses of the patients aged 20 years and older who visited the Ekhlaspur Center of Health: September 1999-August 2001.

Diagnosis	Number (%)		

	Male	Female	Total
Anemia and malnutrition	7 ( 1.5)	39 ( 4.9)	46 ( 3.6)
Anxiety neurosis	6 ( 1.3)	7 ( 0.9)	13 ( 1.0)
Asthma, chronic obstructive pulmonary disease	14 ( 3.0)	10 ( 1.3)	24 ( 1.9)
Common cold	8 ( 1.7)	12 ( 1.5)	20 ( 1.6)
Dysentery, diarrhea	12 ( 2.5)	10 ( 1.3)	22 ( 1.7)
Eczema, dermatitis	7 ( 1.5)	10 ( 1.3)	17 ( 1.3)
Hypertension	19 ( 4.0)	46 ( 5.8)	65 ( 5.1)
Ischaemic heart disease	8 ( 1.7)	7 ( 0.9)	15 ( 1.2)
Leukorrhoea		59 ( 7.4)	59 ( 4.6)
Muskulo-skeletal disorder	10 ( 2.1)	4 ( 0.5)	14 ( 1.1)
Otitis media	8 ( 1.7)	12 ( 1.5)	20 ( 1.6)
Peptic ulcer	58 (12.3)	124 (15.5)	182 (14.3)
Pregnancy		23 ( 2.9)	23 ( 1.8)
Respiratory tact infection	10 ( 2.1)	8 ( 1.0)	18 ( 1.4)
Rheumatic fever, rheumatic heart disease	4 ( 0.8)	10 ( 1.3)	14 ( 1.1)
Rheumatological disorders	47 (10.0)	78 ( 9.8)	125 ( 9.8)
Scabies	14 ( 3.0)	6 ( 0.8)	20 ( 1.6)
Urinary tract infection	17 ( 3.6)	27 ( 3.4)	44 ( 3.5)
Others	109 (23.1)	175 (21.9)	284 (22.3)
Sign, symptoms and ill-defined problem	86 (18.3)	100 (12.5)	186 (14.6)
Missing diagnosis	27 ( 5.7)	33 ( 4.1)	60 ( 4.7)
Total	471 (100)	800 (100)	1271 (100)

*Main diagnosis for which the patients sought medical treatment. Associated conditions/diseases are not shown.

Age, education, and dietary frequency of meat and fish are shown in Table 2. A half of the patients had primary education, but one fourth had no formal education. One of the 4 the patients took meat only once in the last three days on the contrary more than 75% had fish six times in the same period.

**Table 2.** Age, education, and diet of a clinic-based rural population of Bangladesh.

	median (lower quartile - upper quartile)		

	Men (n=471)	Women (n=800)	Total (n=1271)
Age (year)	40 (30-60)	40 (30-60)	38 (28-50)
Education (year)	5 (0-9)	5 (0-9)	5 (0-8)
Dietary frequency (times/last 3 days)			
Mutton, beef, and poultry	0 (0-1)	0 (0-1)	0 (0-1)
Fish	4 (2-6)	4 (2-6)	4 (2-6)

*results are median (lower quartile - upper quartile)

Age- and sex-specific distribution of non-biochemical CVD risk factors are shown in Table 3. Mean body mass index was 18.5 kg/m^2^ in males and 18.7 kg/m^2^ in females, and the highest mean BMI 19.6 kg/m^2^ and 19.2 kg/m^2^ was observed in 30-39 years age group in males and females, respectively. Mean waist circumference was 71.3 cm in males and 68 cm in females. Males of 30’s and 40’s of age had the highest mean waist circumference. Proportion of overweight (BMI: 25.0+ kg/m^2^) was 2.4% in males and 4.3% in females, and that with mild abdominal obesity (waist circumference >94 cm in males, and >80 cm in females) was 1.7 % for males 11.4% for females. Females had higher prevalence of abdominal obesity than males. There were negligible people with higher grades of obesity.

**Table 3.** Non-biochemical risk factors for cardiovascular diseases in clinic-based rural population of Bangladesh.

	Age (year)						Total

	20-29	30-39	40-49	50-59	60-69	70-	
	males						
Number	107	93	90	56	65	60	471
Mean (standard deviation)							
Body mass index (kg/m^2^)	18.5 (2.6)	19.6 (3.6)	19.1 (2.7)	17.8 (2.7)	17.6 (2.7)	17.6 (2.7)	18.5 (2.9)
Waist circumference (cm)	69 (6)	73 (9)	73 (9)	71 (8)	70 (9)	71 (10)	71.3 (8.6)
Systolic blood pressure (mm Hg)	116 (13)	119 (14)	120 (15)	125 (15)	126 (23)	131 (29)	122 (18)
Diastolic blood pressure (mm Hg)	76 (8)	79 (9)	79 (10)	80 (8)	79 (12)	78 (10)	78 (10)
Tobacco use* frequency/day	12 (9)	12 (11)	13 (10)	10 (6)	9 (6)	9 (6)	11 (8)
Percent (standard error)							
Hypertension (140/90+ mmHg)**	7.3 (2.5)	12.8 (3.5)	18.3 (4.1)	21.2 (5.5)	23.0 (5.2)	32.7 (6.1)	17.3 (1.8)
Obesity							
Body mass index 25.0+ kg/m^2^	1.9 (1.3)	6.5 (2.6)	1.1 (1.1)	0.0 (0.0)	3.2 (2.2)	1.7 (1.7)	2.4 (0.7)
Waist 94+ cm	0 (0)	1.1 (1.1)	2.2 (1.5)	1.8 (1.8)	3.2 (2.2)	3.3 (2.3)	1.7 (0.6)
Tobacco user	23.6 (4.0)	38.5 (5.0)	62.9 (5.1)	65.5 (6.4)	53.8 (6.2)	46.7 (6.4)	43.8 (2.2)

	females						
Number	253	191	140	104	74	38	800
Mean (standard deviation)							
Body mass index (kg/m^2^)	18.5 (2.9)	19.2 (3.8)	18.8 (3.3)	18.9 (3.7)	18.2 (3.5)	18.3 (3.0)	18.7 (3.3)
Waist circumference (cm)	67 (9)	69 (10)	68 (10)	68 (10)	68 (9)	69 (11)	68 (10)
Systolic blood pressure (mm Hg)	113 (11)	118 (16)	117 (18)	125 (21)	133 (21)	141 (30)	120 (19)
Diastolic blood pressure (mm Hg)	74 (8)	77 (10)	76 (10)	79 (11)	82 (11)	81 (9)	77 (10)
Tobacco use* frequency/day	6 (4)	7 (5)	9 (6)	9 (6)	8 (6)	5 (3)	8 (6)
Percent (standard error)							
Hypertension (140/90+ mmHg)**	5.7 (1.5)	15.0 (2.6)	17.6 (3.2)	29.8 (4.5)	43.1 (5.8)	45.7 (8.1)	18.2 (1.4)
Obesity							
Body mass index 25.0+ kg/m^2^	2.8 (1.0)	4.7 (1.5)	5.7 (2.0)	9.6 (2.9)	2.8 (1.9)	0 (0)	4.3 (0.7)
Waist 80+ cm	6.4 (1.5)	11.6 (2.3)	12.9 (2.8)	17.5 (3.7)	15.1 (4.2)	18.9 (6.4)	11.4 (1.1)
Tobacco user	4.5 (1.3)	30.0 (3.3)	45.3 (4.2)	37.3 (4.7)	50.7 (5.8)	47.2 (8.1)	27.1 (1.6)

	both						
Number	360	284	230	160	139	98	1271
Mean (standard deviation)	10 (8)	9 (8)	11 (8)	9 (6)	9 (6)	7 (5)	9 (7)
Body mass index (kg/m^2^)	18.5 (2.8)	19.3 (3.7)	18.9 (3.0)	18.5 (3.4)	17.9 (3.1)	17.9 (2.8)	18.7 (3.2)
Waist circumference (cm)	67 (8)	70 (10)	70 (10)	69 (10)	69 (9)	70 (11)	69 (9)
Systolic blood pressure (mm Hg)	114 (12)	118 (15)	118 (17)	125 (19)	130 (22)	135 (30)	120 (19)
Diastolic blood pressure (mm Hg)	74 (8)	77 (10)	77 (10)	79 (10)	81 (12)	79 (10)	77 (10)
Tobacco use* frequency/day	10 (8)	9 (8)	11 (8)	9 (6)	9 (6)	7 (5)	9 (7)
Percent (standard error)							
Hypertension (140/90+ mmHg)**	6.1 (1.3)	14.3 (2.1)	17.8 (2.5)	26.7 (3.5)	33.8 (4.0)	37.8 (4.9)	17.8 (1.1)
Obesity							
Body mass index 25.0+ kg/m^2^	2.5 (0.8)	5.3 (1.3)	3.9 (1.3)	6.3 (1.9)	3.0 (1.4)	1.1 (1.0)	3.6 (0.5)
Waist 94+ or 80+ cm	4.5 (1.1)	8.1 (1.6)	8.7 (1.9)	12.0 (2.6)	9.6 (2.5)	9.3 (2.9)	7.9 (0.8)
Tobacco user	10.2 (1.6)	32.7 (2.8)	52.2 (3.3)	47.1 (3.9)	52.2 (4.2)	46.9 (5.0)	33.4 (1.3)

* : Smoking or chewing among the users.

**: Inclusing anti-hypertensive medication.

Mean SBP and DBP in males were 122 mmHg and 78 mmHg in males, while for females these were 120 mmHg and 77 mmHg, respectively (Table 3). The mean SBP and DBP increased with the increasing age group in both sexes except for DBP in males, which was the highest in 50-59 years of age. The prevalence of hypertension was 17.3% in males and 18.2 % in females. The prevalence of hypertension increased with age.

Prevalence of tobacco consumption (smoking and chewing tobacco) was 33.0% for all subjects, and 43.8 % and 27.1% for males and females, respectively. Among the tobacco users, males used any form of tobacco on average 11 times in a day while females used 8 times in a day. The proportion of tobacco use was higher in older age groups in both males and females (Table 3)

## DISCUSSION

The blood pressure levels that we observed (SBP: 120 mmHg, and DBP: 77 mmHg) are similar to that of our previous study^6^ in another rural population (SBP: 119 mmHg, and DBP: 75 mmHg). Blood pressure levels are also same as those observed in Bangladeshi emigrants, but lower than that in British people.^7^ Prevalence of hypertension in our study (17.8%) is higher than the pooled estimate (11.3%)^1^ and other study (12.9%) in another rural population.^6^ This might be due to the fact that the subjects are clinic-based and any form of illness they had could influence blood pressure.

Use of tobacco leaf for chewing with *pan* particularly by females is a characteristic of our population. Studies have shown that cardiovascular effects of chewable tobacco are similar to those of cigarette smoking,^8^^,^^9^ but the age adjusted relative risk of dying from CVD is lower with smokeless tobacco than tobacco smoking.^10^ We combined cigarette smoking and any form of tobacco use together because reporting only cigarettes smoking would under report the use tobacco in this society. The prevalence of tobacco consumption that we observed (33.4%) is slightly lower than that reported for officials of various ministries (38.6%), an urban population.^5^

Recent data show that waist circumference *per se* is as informative as the waist-hip ratio and less liable to measurement error.^11^ In our survey, more females have central obesity than males defined by waist circumference cut-off points recommended by the World Health Organization^10^ based on Western population. This might be due to the fact that males in this manual agricultural community endure hard physical labor as compared with females. Hard physical labor might be one of the causes of finding of very low prevalence (3.6%) of overweight in this population. Another reason might be that subjects are patients not community living healthy people.

Our data comes from a clinic-based population and may not represent the rural population of Bangladesh. Our results need to be evaluated in the perspective of this limitation. Because the data were collected using a standardized methods and patients from all socio-economic strata of the village with general health problems came to the clinic, we believe that these data may reflect the underlying distribution of risk factors in that community.

We collected only non-biochemical risk factors from all the patients visiting the clinic for consultation. Because it would put extra burden of cost upon patients to measure lipid profile and due to budgetary constrain of this study, we were unable to do the standardized measurement of biochemical risk factors in all patients. Biochemical risk factors such as serum lipids are established CVD risk factors but few studies have looked into the population distribution of blood lipids in Bangladeshis living in native country. A previous study reported that mean serum total cholesterol level in rural middle-aged males was 155.7 mg/dl and that of females 162.0 mg/dl.^6^ Immigrant Bangladeshis living in the United Kingdom have lower high-density lipoprotein cholesterol and higher triglycerides level compared with the Europeans and also with other south Asian populations.^12^ There is also paucity of data regarding prevalence of CVD in Bangladesh. One study showed that the admission for cardiovascular disease is increasing in a government hospital of Dhaka.^13^ Further well-standardized epidemiologic studies in representative population are needed to know the distribution of various blood lipids and also other non-biochemical risk factors and prevalence of CVD in this population. Given that the low population mean of blood cholesterol levels and very low prevalence of obesity and mainly fish and vegetable based diet in rural population, however, tobacco use and hypertension would be the important risk factors for the control of CVD in this population. The situation existing in Bangladeshi rural population might be similar to the Japanese situation of four decades ago in terms of low energy intake from fat, prevalence of hypertension and smoking.^14^^,^^15^ The remarkable achievements of lowering CVD mortality in Japanese population over the last few decades resulting from prevention strategies taken by the Japanese government for control of hypertension, salt intake, and smoking could be followed in this population with culturally appropriate approach. Future collaborative epidemiologic studies between two countries would be beneficial for knowledge transfer and institutional development in the field of prevention of CVD in Bangladesh.
